# Chromosomal stasis in distinct families of marine Percomorpharia from South Atlantic

**DOI:** 10.3897/CompCytogen.11(2).11942

**Published:** 2017-05-04

**Authors:** Fabilene Gomes Paim, Leandro Aragão da Hora Almeida, Paulo Roberto Antunes de Mello Affonso, Patrícia Elda Sobrinho-Scudeler, Claudio Oliveira, Débora Diniz

**Affiliations:** 1 Departamento de Ciências Biológicas, Universidade Estadual do Sudoeste da Bahia (UESB), Jequié, Bahia, Brasil; 2 Laboratório de Biologia e Genética de Peixes, Instituto de Biociências de Botucatu, UNESP, Botucatu, SP, Brasil

**Keywords:** Cytotaxonomy, Ephippiformes, ribosomal genes, South Atlantic

## Abstract

The weakness of physical barriers in the marine environment and the dispersal potential of fish populations have been invoked as explanations of the apparent karyotype stasis of marine Percomorpha, but several taxa remain poorly studied cytogenetically. To increase the chromosomal data in this fish group, we analyzed cytogenetically three widespread Atlantic species from distinct families: *Chaetodipterus faber* Broussonet, 1782 (Ephippidae), *Lutjanus synagris* Linnaeus, 1758 (Lutjanidae) and *Rypticus randalli* Courtenay, 1967 (Serranidae). The three species shared a karyotype composed of 2n=48 acrocentric chromosomes, single nucleolus organizer regions (NORs) and reduced amounts of centromeric heterochromatin. A single NOR-bearing pair was identified in all species by physical mapping of 18S rDNA while non-syntenic 5S rRNA genes were located at centromeric region of a single pair. The similar karyotypic macrostructure observed in unrelated groups of Percomorpharia reinforces the conservative karyoevolution of marine teleosteans. Nonetheless, the species could be differentiated based on the pair bearing ribosomal cistrons, revealing the importance of microstructural analyses in species with symmetric and stable karyotypes.

## Introduction

Perciformes have long been regarded as the largest order of vertebrates with nearly 10.000 species, 1540 genera, and 160 families, most of them inhabiting the marine environment (Nelson 2006, Helfman et al. 2009). Recently, robust molecular studies resolved their phylogenetic uncertainties by placing this and other Percomorpha representatives into 11 orders within a new supraordinal group called Percomorpharia, even though Perciformes remained as the most species-rich order (Betancur et al. 2013). Nonetheless, in spite of their ecological and evolutionary relevance, the marine representatives from this large fish group remain poorly studied from a cytogenetic viewpoint (Galetti et al. 2006) when compared to typical freshwater families (Balen et al. 2013, Cardoso et al. 2013, Gouveia et al. 2013).

In general, chromosomal studies in marine Percomorpharia reveal stable karyotypes composed of 2n=48 and a predominance of acrocentric pairs. Indeed, the presence of 24 acrocentric pairs is shared by several species from distinct families of Perciformes (Affonso et al. 2001, Accioly and Molina 2007, 2008, Cipriano et al. 2008, Motta Neto et al. 2011a, 2011b, 2012, Molina et al. 2012, Molina et al. 2013, Costa et al. 2016). This pattern raises some intriguing questions: (1) how could such a morphologically diversified group evolve without significant chromosomal changes (Brum 1996, Galetti et al. 2000, Molina 2007)? (2) What are the advantages (if any) of maintaining stable karyotypes?

One of the hypotheses invoked to explain the conserved karyoevolution of this fish group refers to their biological traits, such as the absence or fragility of physical barriers in oceans that favor the connectivity among populations, wide range of most species and chromosomal or genomic peculiarities (Molina 2007). In fact, freshwater families of Percomorpha, like Cichlidae, are characterized by higher karyotype variation than marine groups, corroborating the role of allopatric evolution in the process of chromosome differentiation (Brum and Galetti 1997, Feldberg et al. 2003).

On the other hand, most families of marine Percomorpharia have been divided into two groups based on the rate of karyotype changes, comprising families of high karyotype stability or with moderate rates of karyoevolution (Molina et al. 2014). Nonetheless, several species and families lack basic cytogenetic information and refined analyses of chromosomal microstructure are particularly rare in marine fish, thus restraining evolutionary inferences and the extent of their conservative karyoevolution.

To test the corollary that the high dispersal and gene flow associated with the weakness of geographic barriers accounts for the chromosomal stasis in marine Percomorpharia groups, we analyzed cytogenetically three widespread Atlantic species from distinct families: *Chaetodipterus faber* (Ephippidae), *Lutjanus synagris* (Lutjanidae) and *Rypticus randalli* (Serranidae). Besides inferring their karyoevolutionary pathways, we provided the first cytogenetic report in *C. faber* and *R. randalli*.

## Methods

The specimens of *Chaetodipterus faber* (N=7, 1♀, 6 unidentified sex), *Lutjanus synagris* (N=8, 4♀, 4 unidentified sex), and *Rypticus randalli* (N=10, 3♂, 2♀, 5 unidentified sex) were collected by gillnets and snorkeling in Camamu Bay and Boipeba Island, located on the coast of Bahia, northeastern Brazil, South Atlantic. The vouchers were deposited in the Laboratory of Genetics of Aquatic Organisms (LAGOA) from Universidade Estadual do Sudoeste da Bahia, in Jequié, Bahia.

After collection, the specimens were mitotically stimulated by inoculation of fungal antigens and kept in fish tanks for 24 to 72 h (Lee and Elder 1980). After euthanasia in iced water (Blessing et al. 2010), the anterior kidney was removed and used to obtain mitotic chromosomes (Netto et al. 2007, Blanco et al. 2012). These procedures were approved by the Committee of Animal Ethics (CEUA/UESB) from Universidade Estadual do Sudoeste da Bahia (71/2014).

The heterochromatin regions were visualized by C-banding (Sumner 1972) while active nucleolus organizer regions (NORs) were detected by silver nitrate staining (Howell and Black 1980). The sequences of 18S and 5S rRNA genes were mapped simultaneously onto chromosomes by double fluorescence *in situ* hybridization (FISH) with a stringency of 77% (Pinkel et al. 1986). The 18S and 5S ribosomal sequences were obtained via polymerase chain reaction (PCR) using samples of genomic DNA of *Moenkhausia sanctaefilomenae* and labeled with16-dUTP–biotin and digoxigenin-11-dUTP (Roche®), respectively. The signal detection was accomplished by using fluorescein isothiocyanate-avidin conjugate (Sigma-Aldrich®) for 18S and anti-digoxigenin-Rhodamine (Roche®) for 5S rDNA. The chromosomes were counterstained using 4'6-diamidino-2-phenylindole (DAPI) at 0.2 mg/mL in Vectashield Mounting Medium (Vector®).

The metaphase spreads were photographed using an epifluorescence microscope Olympus BX-51 equipped with the software ImagePro-Plus v. 6.2. (Media Cybernetics).The chromosomes were classified according to their arm ratio (Levan et al. 1964) and organized into pairs by decreasing size order in karyotypes.

## Results

**Figure 1.**
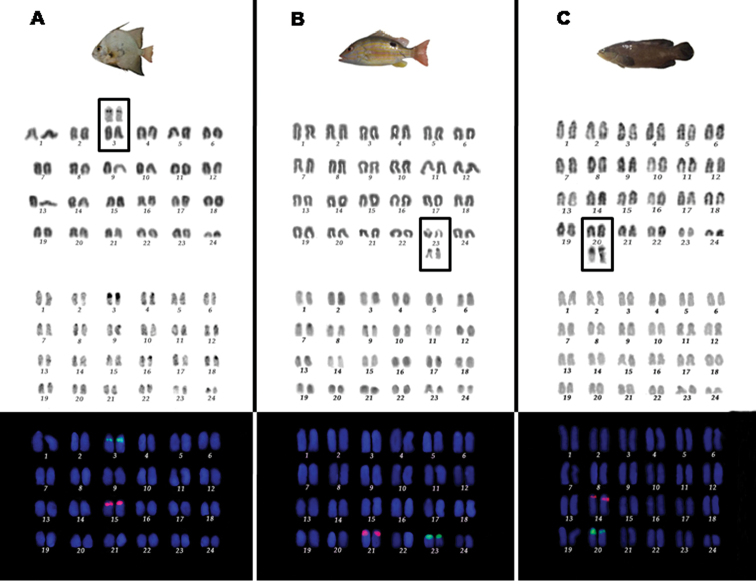
Karyotypes of *Chaetodipterus faber* (**A**), *Lutjanus synagris* (**B**), and *Rypticus randalli* (**C**) with 2n=48 acrocentric chromosomes after conventional Giemsa-staining (top), C-banding (center) and double FISH with 18S (green signals) and 5S (magenta signals) rDNA probes (bottom). In boxes, the pairs bearing nucleolus organizer regions after silver nitrate staining (Ag-NORs).

The species *C. faber*, *L. synagris*, and *R. randalli* share a modal diploid number of 2n = 48, composed exclusively of acrocentric chromosomes (Figure 1). The heterochromatin distribution is reduced, being located at centromeric or pericentromeric regions of most chromosomes in the three species (Figure 1A, B, C). Particularly, *C. faber* showed conspicuous heterochromatic blocks in pair 3, being coincident with NORs (Figure 1A, box).

Single NORs were invariably detected, but located at distinct pairs according to each species (Figure 1A, 1B, 1C, Box). The NORs in *C. faber* were located at interstitial position on long arms of pair 3 (Figure 1A, box). On the other hand, the NOR-bearing pair corresponds to the 23^rd^ pair in *L. synagris*, with marks at interstitial region close to centromeres, in agreement with secondary constrictions revealed by Giemsa-staining (Figure 1B, box). In *R. randalli*, the NORs were detected at pericentromeric region of pair 20, being characterized by size heteromorphism between homologous (Figure 1C, box).

The 18S rDNA sites were located at interstitial positions on the long arms of pairs 3, 20, and 23 in *C. faber*, *R. randalli*, and *L. synagris*, respectively. Size differences in the 18S rDNA clusters between homologs were observed in *C. faber* and *R. randalli*, as also revealed by silver nitrate staining (Figure 1A, 1B, 1C). The 5S rDNA sequences were mapped at pericentromeric region on long arms of all studied species, corresponding to the pairs 15 in *C. faber*, 21 in *L. synagris* and 14 in *R. randalli* (Figure 1A, 1B, 1C).

## Discussion

The three species studied in the present work shared a karyotype composed of 24 pairs of acrocentric chromosomes, regarded as a plesiomorphic feature for Perciformes sensu Nelson 2006 (Brum 1996, Galetti et al. 2006, Molina 2007, Arai 2011) in spite of the derived position of some representatives in phylogenetic studies. Indeed, according to the recent classification of bony fish, *C. faber* (Ephippidae) would belong to a distinct order (Ephippiformes) while Lutjanidae has been placed apart from other Perciformes families (e.g. Serranidae) within Percomorpharia (Betancur et al. 2013). Indeed, this symplesiomorphic karyotype has been commonly reported in serranids (groupers and allies) (Molina et al. 2002), just like herein described for *R. randalli*.

Likewise, lutjanids (snappers) from the Brazilian coast invariably present 2n=48 acrocentric chromosomes (Rocha and Molina 2008), as corroborated by our data in *L. synagris*. However, cytogenetic studies in Caribbean populations of *L. synagris* revealed an additional karyomorph with 2n=47 (1 metacentric and 46 acrocentric chromosomes), characterizing a polymorphic condition (Nirchio et al. 2008).

The karyotypic results in *C. faber* represent the first cytogenetic data in the order Ephippiformes (Betancur et al. 2013), which constrains inferences about chromosomal evolution in this group. Nonetheless, the karyotype macrostructure of this species follows the common trend observed in most Percomorpharia groups (e.g. Haemulidae, Scianidae, Lutjanidae and Serranidae) (Nirchio et al. 2014).

Besides the role of dispersal and formation of large populations (Molina 2007), some authors have inferred that speciation driven mainly by ecological features rather than by genetic isolation *per se* could result in a high number of species with similar karyotypes, as proposed for Haemulidae and Lutjanidae (Rocha and Molina 2008, Motta Neto et al. 2012). Moreover, intrinsic genome features could favor a conserved karyoevolution in these marine fish families. In common, most of studied species with basal karyotypes are poor in heterochromatin content and other repetitive sequences, which have been associated with the dynamics and rates of chromosomal changes (Molina 2007, Costa et al. 2013). However, detailed studies of karyotype microstructure are scarce for most marine fish species. Thus, microstructural chromosomal changes not affecting the number and morphology of chromosomes could remain undetected, misleading to the apparent chromosomal stability in Perciformes and allies (Nirchio et al. 2014). Therefore, chromosomal studies including banding methods and mapping of specific DNA sequences, as carried out in the present study, are particularly important to infer the karyotype structure of Percomorpharia and the evolutionary forces that could determine interspecific variation in spite of the conservativeness of karyotype macrostructure.

The nucleolus organizer regions (NORs) are considered a highly informative cytogenetic marker in teleosteans (Gornung 2008). The presence of single NORs at pericentromeric region is considered the plesiomorphic condition for several families in Percomorpharia, particularly those characterized by species with 2n=48a (Affonso et al. 2001, Motta Neto et al. 2011b, Molina et al. 2013), as also supported by the present results of 18S rDNA FISH and silver nitrate staining. In the case of *L. synagris*, the pattern of distribution of ribosomal genes was similar to that previously described for other populations in Brazil (Costa et al. 2016). Similarly, NOR size heteromorphism between homologous chromosomes in other fish species bearing single 18S rDNA clusters (Foresti et al. 1981) is widespread, as also detected in this report. Usually, this polymorphism is related to spontaneous duplications/deletions or unequal crossover between homologous (Affonso et al. 2002).

On the other hand, the NOR-bearing pair seems to differ according to each species in some families, like Lutjanidae (Costa et al. 2016) and Serranidae (Molina et al. 2002). Accordingly, in spite of sharing the same karyotype macrostructure and the number of 18S rDNA sites, the three species herein studied could be distinguished by the NOR-bearing pair (3 in *C. faber*, 23 in *L. synagris*, and 20 in *R. randalli*). Even though the precise establishment of pairs is hindered by the subtle size differences of acrocentric chromosomes, thereby being susceptible to some degree of subjectivity, the NOR-bearing pairs in the species clearly belong to distinctive categories according to size, ranging from large (*C. faber*) to small (*L. synagris*) pairs.

The identification of 5S rRNA genes was also informative to the karyotypic analyses of studied species. As commonly reported in marine fish, particularly Perciformes (Motta Neto et al. 2011a, Martins et al. 2011, Molina et al. 2013), the 5S rDNA clusters were non-syntenic to NORs and located close to centromeres, revealing their independent evolution in relation to 18S rDNA. Apparently, this trend is widespread in Percomorpharia once it was identified in distinct orders according to the recent phylogenetic tree of teleosteans (Betancur et al. 2013). As observed for 18S rDNA, the pairs carrying 5S rDNA clusters also differed among each species suggesting they represent species-specific markers, even though the pairs were more similar in size (15 in *C. faber*, 21 in *L. synagris*, and 14 in *R. randalli*).

In conclusion, the present results highlight the extensive karyotype macrostructure stasis in marine Percomorpha, since several cytogenetic features were shared by three species from distinct families and groups within Percomorpharia, corroborating the hypothesis of conserved karyotype macrostructure in widespread marine species. However, the evolutionary dynamics of ribosomal genes seem to play a major role in the cytotaxonomy of marine fish, as pointed out in typical marine families with basal karyotypes like Lutjanidae (Costa et al. 2016). Therefore, the mapping of distinct classes of repetitive DNA is highly recommended to provide a reliable scenario about the chromosomal evolution of groups with apparent stable karyotypes, as Perciformes and their allies.

## References

[B1] Accioly IV Molina WF (2008) Cytogenetic studies in Brazilian marine Sciaenidae and Sparidae fishes (Perciformes). Genetics and Molecular Research 7(2): 358–370. https://doi.org/10.4238/vol7-2gmr427 18551402 10.4238/vol7-2gmr427

[B2] Accioly IV Molina WF (2007) Contribuição à citogenética dos gêneros *Pomadasys* e *Anisotremus* (Haemuldae, Perciformes). PublIca III: 36–44. http://periodicos.ufrn.br/publica/article/view/109/105

[B3] Affonso PRAM Guedes W Pauls E Galetti PM Jr. (2001) Cytogenetic Analysis of Coral Reef Fishes from Brazil (Families Pomacanthidae and Chaetodontidae). Cytologia 66: 379–384. http://doi.org/10.1508/cytologia.66.379

[B4] Affonso PRAM Guedes W Pauls E Galetti PM Jr. (2002) Close karyotypical relationship between two species of marine angelﬁshes from South Atlantic: *Pomacanthus arcuatus* and *P. paru* (Perciformes, Pomacanthidae). Caryologia 55(4): 323–329. http://dx.doi.org/10.1080/00087114.2002.10797883

[B5] Arai R (2011) Fish Karyotypes: A Check List. Springer, Japan, 340 pp https://doi.org/10.1007/978-4-431-53877-6

[B6] Blanco DR Bertollo LAC Lui RL Vicari MR (2012) A new technique for obtaining mitotic chromosome spreads from fishes in the field. Journal of fish biology 81(1): 351–7. https://doi.org/10.1111/j.1095-8649.2012.03325.x 22747826 10.1111/j.1095-8649.2012.03325.x

[B7] Balen RE Noleto RB Vicari MR Artoni RF Cestari MM (2013) Comparative cytogenetics among populations of *Hollandichthys multifasciatus* (Teleostei: Characidae). Zoological science 30(2): 105–9. http://dx.doi.org/10.2108/zsj.30.105 23387844 10.2108/zsj.30.105

[B8] Betancur RR Broughton RE Wiley EO Carpenter K López JA Li C (2013) The tree of life and a new Classification of bony fishes. PLOS Currents Tree of Life, 49 pp https://doi.org/10.1371/currents.tol.53ba26640df0ccaee75bb165c8c26288 10.1371/currents.tol.53ba26640df0ccaee75bb165c8c26288 PMC3644299 23653398

[B9] Blessing JJ Marshall JC Balcombe SR (2010) Humane killing of fishes for scientific research: a comparison of two methods. Journal of Fish Biology 76: 2571–2577. https://doi.org/10.1111/j.1095-8649.2010.02633.x 20557609 10.1111/j.1095-8649.2010.02633.x

[B10] Brum MJI (1996) Cytogenetics studies of Brazilian marine fish. Brazilian Journal of Genetics 19(3): 421–427.

[B11] Brum MJI Galetti PM Jr. (1997) Teleostei ground plan karyotype. Journal of Computational Biology 2: 91–102.

[B12] Cardoso AL Sales KAH Nagamachi CY Pieczarka JC Noronha RCR (2013) Comparative cytogenetics of two species of genus *Scobinancistrus* (Siluriformes, Loricariidae, Ancistrini) from the Xingu River, Brazil. Comparative cytogenetics 7(1): 43–51. https://doi.org/10.3897/CompCytogen.v7i1.4128 24260689 10.3897/CompCytogen.v7i1.4128 PMC3833746

[B13] Cipriano RR Fenocchio AS Artoni RF (2008) Chromosomal Studies of Five Species of the Marine Fishes From the Paranaguá Bay and the Karyotypic Diversity in the Marine Teleostei of the Brazilian Coast. Brazilian Archives of Biology and Technology 51(April): 303–314. http://dx.doi.org/10.1590/S1516-89132008000200010

[B14] Costa GWWF Cioffi MB Bertollo LAC Molina WF (2016) The Evolutionary Dynamics of Ribosomal Genes, Histone H3, and Transposable Rex Elements in the Genome of Atlantic Snappers. Journal of Heredity 107(2): 173–180. https://doi.org/10.1093/jhered/esv136 26792596 10.1093/jhered/esv136 PMC5994970

[B15] Costa GWWF Cioffi MB Bertollo LAC Molina WF (2013) Transposable elements in fish chromosomes: A Study in the marine Cobia species. Cytogenetic Genome Research 1–7. https://doi.org/10.1159/000354309 10.1159/000354309 23969732

[B16] Feldberg E Porto JIR Bertollo LAC (2003) Chromosomal changes and adaptation of cichlid fishes during evolution. In: Val AL Kapoor BG (Eds) Fish Adaptation. IBH & Oxford, New Dehli & New York, 287–310.

[B17] Foresti F Almeida–Toledo LF Toledo–Filho AS (1981) Polymorphic nature of nucleolus organizer regions in fishes. Cytogenet Cell Genetics 31: 137–144. https://doi.org/10.1159/000131639 10.1159/000131639 6173166

[B18] Galetti PM Jr. Aguilar CT Molina WF (2000) An overview of marine fish cytogenetics. Hydrobiologia 420(1): 55–62. https://doi.org/10.1023/A:1003977418900

[B19] Galetti PM Jr. Molina WF Affonso PRAM Aguilar CT (2006) Assessing genetic diversity of Brazilian reef fishes by chromosomal and DNA markers. Genetica 126: 161–177. https://doi.org/10.1007/s10709-005-1446-z 16502093 10.1007/s10709-005-1446-z

[B20] Gouveia JG Moraes VPO Pires LB Rosa R Dias AL (2013) Comparative cytogenetics between two species of the family Pseudopimelodidae (Siluriformes): occurrence of natural triploidy and supernumerary chromosomes. Cytotechnology 67(2): 215–222. https://doi.org/10.1007/s10616-013-9676-x 24363190 10.1007/s10616-013-9676-x PMC4329307

[B21] Gornung E (2013) Twenty years of physical mapping of major ribosomal RNA genes across the teleosts: A review of research. Cytogenetic and genome research 141(2–3): 90–102. https://doi.org/10.1159/000354832 24080951 10.1159/000354832

[B22] Helfman GS Collette BB Facey DE Bowen BW (2009) The Diversity of Fishes. Wiley-Blackwel.

[B23] Howell WM Black DA (1980) Controlled silver staining of nucleolus organizer region with protective colloidal developer: a 1-step method. Experientia 36: 1014–1015. https://doi.org/10.1007/BF01953855 6160049 10.1007/BF01953855

[B24] Lee MR Elder FFB (1980) Yeast stimulation of bone marrow mitosis for cytogenetic investigations. Cytogenetics Cell Genetics 26: 36–40. https://doi.org/10.1159/000131419 6989561 10.1159/000131419

[B25] Levan A Fredga K Sandberg AA (1964) Nomenclature for centromeric position on chromosomes. Hereditas 52: 201–220. https://doi.org/10.1111/j.1601-5223.1964.tb01953.x

[B26] Martins C Cabral-de-Mello DC Valente GT Mazzuchelli J Oliveira SG (2011) Cytogenetic mapping and its contribution to the knowledge of animal genomes. In: Kevin VU (Ed.) Advances in Genetics Research, Vol. 4. Nova Science Publisher, Hauppauge, NY, 1–82.

[B27] Molina WF Martinez PA Bertollo LAC Bidau CJ (2014) Preferential accumulation of sex and Bs chromosomes in biarmed karyotypes by meiotic drive and rates of chromosomal changes in fishes. Anais da Academia Brasileira de Ciências 86(4): 1801–1812. doi: http://dx.doi.org/10.1590/0001-3765201420130489 25590717 10.1590/0001-3765201420130489

[B28] Molina WF Costa GWWF Soares RX Affonso PRAM (2013) Extensive chromosome conservatism in Atlantic butterflyfishes, genus *Chaetodon* Linnaeus, 1758: Implications for the high hybridization success. Zoologischer Anzeiger – A Journal of Comparative Zoology 253(2): 137–142. http://dx.doi.org/10.1016/j.jcz.2013.10.001

[B29] Molina WF Motta Neto CC Sena DCS Cioffi MB Bertollo LAC (2012) Karyoevolutionary aspects of Atlantic hogfishes (Labridae-Bodianinae), with evidence of an atypical decondensed argentophilic heterochromatin. Marine genomics 6: 25–31. http://dx.doi.org/10.1016/j.margen.2012.01.001 22578656 10.1016/j.margen.2012.01.001

[B30] Molina WF (2007) Chromosome changes and stasis in marine fish groups. In: Pisano E Ozouf-Costaz C Foresti F Kapoor BG (Eds) Fish Cytogenetic. CRC Press, Boca Raton (FL), 69–110.

[B31] Molina WF Maia-Lima FA Affonso PRAM (2002) Divergence between karyotypical pattern and speciation events in Serranidae fish (Perciformes). Caryologia 55(4): 299–305. http://dx.doi.org/10.1080/00087114.2002.10797880

[B32] Motta Neto CC Cioffi MB Bertollo LAC Molina WF (2011a) Extensive chromosomal homologies and evidence of karyotypic stasis in Atlantic grunts of the genus *Haemulon* (Perciformes). Journal of Experimental Marine Biology and Ecology 401(1–2): 75–79. http://dx.doi.org/10.1016/j.jembe.2011.02.044

[B33] Motta Neto CC Cioffi MB Bertollo LAC Molina WF (2011b) Molecular cytogenetic analysis of Haemulidae fish (Perciformes): Evidence of evolutionary conservation. Journal of Experimental Marine Biology and Ecology 407(1): 97–100. http://dx.doi.org/10.1016/j.jembe.2011.07.014

[B34] Motta Neto CC Lima-Filho PA Araújo WC (2012) Differentiated evolutionary pathways in Haemulidae (Perciformes): karyotype stasis versus morphological differentiation. Reviews in Fish Biology and Fisheries 22(2): 457–465. https://doi.org/10.1007/s11160-011-9236-4

[B35] Nelson JS (2006) Fishes of the World. John Wiley & Sons, 624 pp.

[B36] Netto MRCB Pauls E Affonso PRAM (2007) A standard protocol for obtaining fish chromosomes under post-mortem conditions. Micron 38: 214–217. https://doi.org/10.1016/j.micron.2006.07.019 16979897 10.1016/j.micron.2006.07.019

[B37] Nirchio M Rondón R Oliveira C (2008) Cytogenetic studies in three species of *Lutjanus* (Perciformes: Lutjanidae: Lutjaninae) from the Isla Margarita, Venezuela. Neotropical Ichthyology 6(1): 101–108. http://dx.doi.org/10.1590/S1679-62252008000100012

[B38] Nirchio M Rossi AR Foresti F Oliveira C (2014) Chromosome evolution in fishes: a new challenging proposal from Neotropical species. Neotropical Ichthyology 12(4): 761–770. http://dx.doi.org/10.1590/1982-0224-20130008

[B39] Pinkel D Straume T Gray JW (1986) Cytogenetic analysis using quantitative high-sensitivity, fluorescence hybridization. Proceedings of the National Academy of Sciences 83: 2934–2938. 10.1073/pnas.83.9.2934 PMC323421 3458254

[B40] Rocha EC Molina WF (2008) Cytogenetic analysis in western Atlantic snappers (Perciformes, Lutjanidae). Genetics and Molecular Biology 31(2): 461–467. http://dx.doi.org/10.1590/S1415-47572008000300011

[B41] Sumner AT (1972) A simple technique for demonstrating centromeric heterochromatin. Experimental CellResearch 75: 304–306. https://doi.org/10.1016/0014-4827(72)90558-7 10.1016/0014-4827(72)90558-7 4117921

